# In vivo through-range passive stiffness of the lumbar spine: a meta-analysis of measurements and methods

**DOI:** 10.1007/s11517-022-02609-w

**Published:** 2022-07-01

**Authors:** Andrew A. Watt, Andrew J. Callaway, Jonathan M. Williams

**Affiliations:** Faculty of Health and Social Sciences, Bournemouth University, Bournemouth, Dorset UK

**Keywords:** Trunk, Low back, Biomechanics, Moment, Review

## Abstract

Passive spinal stiffness is an important property thought to play a significant role in controlling spinal position and movement. Measuring through-range passive stiffness in vivo is challenging with several methods offered in the literature. Currently, no synthesis of values or methods exists to which to compare literature to. This study aims to provide a contemporary review and quantitative synthesis of the through-range in vivo passive lumbar spinal stiffness values for each of the cardinal planes of movement. A structured systematic search, following PRISMA guidelines, of 28 electronic databases was conducted in 2022. Articles were restricted to peer-reviewed English language studies investigating in vivo through-range passive stiffness of the lumbar spine. Thirteen studies were included, ten relating to flexion/extension, four to lateral bending and five to axial rotation. Average stiffness values, as weighted means and confidence intervals, for each of the four sections of the moment-movement curves were synthesised for all planes of movement. Lateral bending was found to be the comparatively stiffest movement followed by flexion and then axial rotation. Future research should focus on the validity and reliability of measurement techniques. Axial rotation would also benefit from further study of its latter stages of range.

**Graphical abstract:**

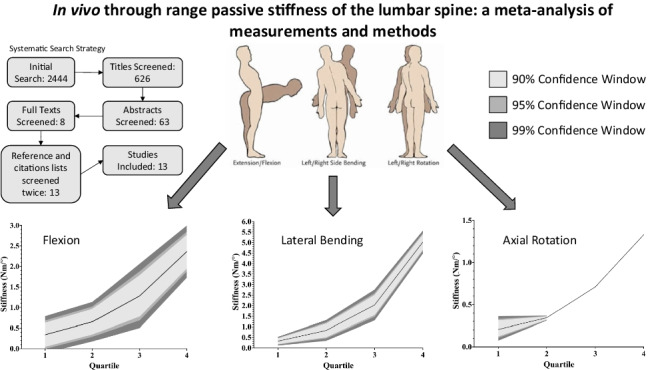

## Introduction

Low back pain is one of the leading causes of disability globally [24]. Despite the large amount of research investigating the spine, much is still not understood of its basic biomechanics [11]. In Panjabi’s [12, 13] seminal articles, it was hypothesised that the spine is controlled through three distinct but closely interwoven systems, the passive, active and neurological control systems, and that dysfunction in any of these systems could lead to pathological changes or pain. Within the passive control system, the characteristic of stiffness has been of great interest within the literature. Stiffness, in a mechanical sense, is a tissue or structure’s ability to resist deformation when loaded [1]. When considering that a function of many of the passive structures in the spine, such as the intervertebral discs and ligaments, is to limit excessive joint motion, it is clear why this tissue property is of great interest and importance.

Spinal stiffness has historically been measured in vitro [11], predominantly by studying functional spinal units, a spinal specimen consisting of two adjacent vertebrae and the connecting disc, ligaments and facet joints. Despite the clear value studies such as these offer, they are inherently limited when attempting to describe the behaviour of the in vivo spine with all its added anatomical complexity. This in vivo behaviour is of great importance, especially for professions utilising manual therapies, including but not limited to physiotherapy and chiropractic, as well as in fields such as biomedical engineering. Within these fields and the surrounding literature, in vivo stiffness measurement techniques have been developed where posteroanterior forces are applied to the spine of an individual lying prone and, from knowing the force and the displacement of the spine, the stiffness can be derived [16, 23].

Although these techniques provide valuable information, they can only describe the resistance of the spine (including the soft tissues) to applied posteroanterior load and not the through-range stiffness properties of the lumbar spine as a whole. Therefore, this single measure of stiffness is unable to report stiffness through range. This limitation restricts the utility of these measurements and may be the reason the link between a single-point estimate of stiffness and loss of function is currently minimal. Techniques for through-range stiffness assessment do exist, being first implemented by McGill et al. [10]. The value of these techniques over others is that they can investigate the stiffness properties of the spine in vivo during physiological movements, rather than simply in one position, and can quantify the stiffness properties throughout the movement, rather than just at one static point in the movement.

Since McGill et al. [10] first measured through-range stiffness, it has been utilised in a wide variety of applications [8, 9, 19]; however, no study to date has provided a quantitative synthesis of the baseline through-range in vivo passive stiffness characteristics of the lumbar spine. This important foundational knowledge around in vivo stiffness would provide reference values for future studies. The aim of this study is to systematically examine two key areas: firstly, to synthesise the average through-range in vivo stiffness of the lumbar spine in the three cardinal planes of movement, completing a quantitative synthesis of the numbers to determine best estimates of stiffness from the included literature. and secondly, to identify methodological differences which may influence the variability of the results to provide a better understanding of these techniques enabling future research to draw on these findings to implement robust methodologies.

## Method

### Search strategy

A search of electronic databases (Medline, SPORTDiscus, CINAHL, Science Direct, Academic Search Ultimate, Complementary Index, Science Citation Index, OAIster, Supplemental Index, Academic Search Complete, British Library Document Supply Centre Inside Serials & Conference Proceedings, Directory of Open Access Journals, Networked Digital Library of Theses and Dissertations, ClinicalTrials.gov, J-STAGE, APA PsycInfo, OpenDissetations, IEEE Xplore Digital Library, SciELO, Environment Complete, JSTOR Journals, SwePub, Business Source Limited, Education Source, British Library EThOS, SocINDEX, Open Research Library, Digital Access to Scholarship at Harvard and Bournemouth University Research Online) was conducted in February 2022. The Boolean search terms used for this search can be seen in Table 1. The search was limited to peer-reviewed, English language journal articles.

**Table 1:** Search terms

Boolean function	Location	Search terms
	Title	spine OR spinal OR trunk OR “low back” OR “lower back” OR “low-back” OR “lower-back” OR vertebral OR vertebrae OR torso OR core
AND	Any	stiffness OR stiff OR resist OR resistance OR resisting OR rigidity
AND	Any	lumbar OR thoracolumbar OR sacrolumbar
NOT	Any	cadaver* OR “in vitro” OR “in-vitro” OR canine* OR rat* OR mouse OR cat* OR dog* OR calf* OR porcine* OR equine* OR sheep OR feline* OR bovine* OR pig* OR mice

**Fig. 1:**
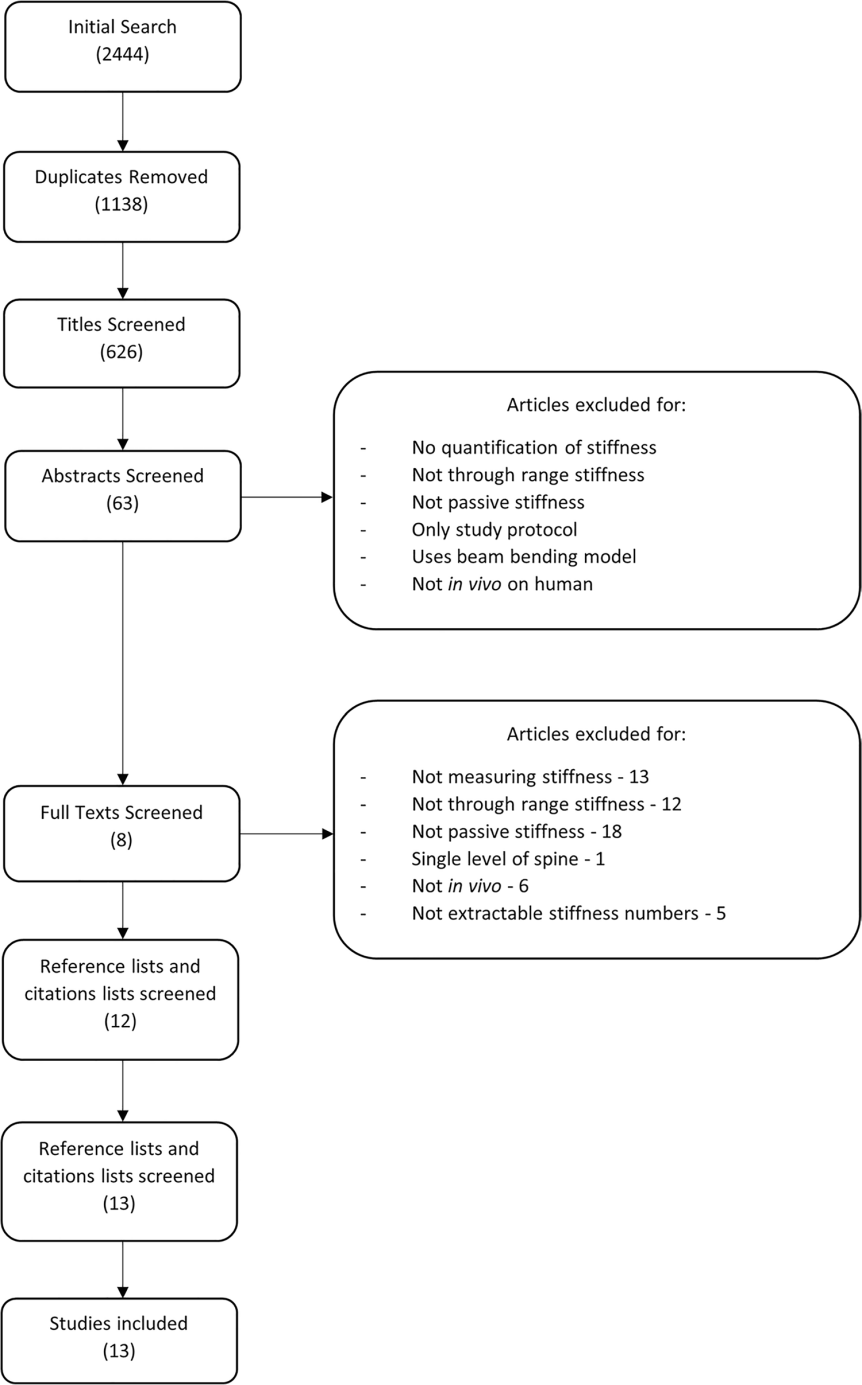
PRISMA diagram. Bracketed numbers represent the number of articles still for inclusion after each stage

Duplicates were removed, and the remaining articles were reviewed by title against the inclusion and exclusion criteria described below. After an initial screening of abstracts, two authors independently reviewed the remaining articles by their abstracts and those not excluded had their full texts reviewed. Any uncertainty around inclusion was resolved by consensus. The articles qualifying for inclusion had their reference lists reviewed for titles that may be relevant, and any articles that appeared potentially applicable were reviewed in the same fashion as the titles found in the original search. In addition, studies in which the included articles were cited were identified via Google Scholar and were reviewed in the same format as for the reference lists. This process was then iterated a further time for the newly included articles. A flow diagram depicting the complete search process can be seen in Fig. 1.

### Inclusion/exclusion criteria

For articles to be included, they were required to be studying the through-range passive stiffness of the lumbar spine in living humans. To that effect, purely cadaveric, computational or animal studies were all excluded. Studies only investigating stiffness in contrast to physiological movements were excluded such as those utilising a beam bending model. As the aim was to calculate average stiffness values of the lumbar spine in non-elderly adults, it was important to limit the effects of aging as best as possible; therefore, the upper age limit for the average age of study participants was set at 60 years. If the average age was above 60 for the study but there was a separated-out cohort with an average age below 60, then the study was included, but only the data for the cohort whose average age was below 60 was included in the synthesis. To ensure only adults were included, the lower age limit of participants was set as 18 years. As the primary aim of this study was to synthesise numerical values of stiffness, articles were required to include numerical values of stiffness or variables that could be used to calculate stiffness. Therefore, studies that only presented their results in graphical form were excluded. In addition, those studies whose stiffness calculations were not clearly explained were excluded.

### Data extraction

As the studies varied in method and topic of investigation, only some of the data provided was relevant to this study. Due to the purpose of this review being to identify the normal values of spinal stiffness in the adult population, only data from suitably aged (see above) control participants or participants pre-study activity/intervention had their data extracted for reporting. In addition to this, where studies investigated differences in cohorts, such as those with back pain compared to controls, only the control groups were used, as defined by the studies themselves.

Where studies presented their results split into groups such as for male and female, the values were averaged between the two groups to find the mean for the cohort. This was also done where studies investigated lateral bending or rotation and presented their data split into left- and right-sided movements.

### Synthesis of stiffness values

**Fig. 2:**
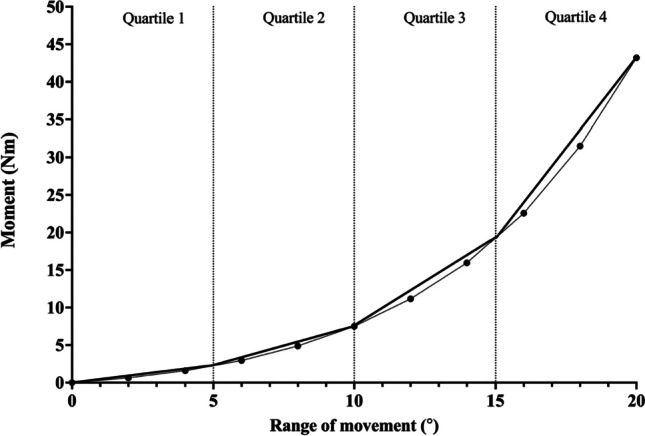
Example moment vs range of movement graph split into quartiles produced from values reported by McGill et al. [10]. Average stiffness values represented by gradient of line of best fit for each quartile. Nm, Newton meter

Due to the heterogeneity of the methodology and results reported in each study, this study utilised the methods developed by McGill et al. [10] to achieve a quantitative synthesis of average stiffness values for each quartile of the moment-range of movement (ROM) relationship (example of quartiles in Fig. 2).

Where multiple studies presented results in the same or comparable units, the results were collated and, where possible, presented in quartiles of range. Some studies originally presented their results as such but for those that did not, their results were used to calculate results for these quartiles to facilitate comparison. Both the raw results for these studies and their quartile results calculated by the authors of this study are presented in Tables 2, 3 and 4.

**Table 2:** Data extraction table for studies investigating flexion/extension

Author and reference	Participants	Methods for stiffness measurements	Stiffness results
Beach et al. [2]	• 12 participants • 6 female and 6 male • Female (averages): ○ Age: 23.3 (1.8) years ○ Height: 1.62 (0.06) m ○ Mass: 58.6 (7.0) kg • Male (averages): ○ Age: 24.5 (1.9) years ○ Height: 1.77 (0.07) m ○ Mass: 76.8 (15.0) kg	• Assessing flexion and extension in side-lying • Participant lying on side on two separate platforms • Legs (pelvis down) fixed to static platform and torso on ‘frictionless’ moveable platform • Lumbar spine movements measured using electromagnetic tracking system with the source placed on the sacrum and the sensor placed over the spinous process of L1 • Electromyography (EMG) electrodes placed on erector spinae muscle bellies bilaterally at T9 and L3 level • Passivity considered EMG amplitude less than 5% of maximum voluntary contraction (MVC) • Participant’s torso was pulled using a perpendicular force to the line of the body applied to the top of the torso platform, measured by a force transducer attached to the pulling cable • Time synchronised force and lumbar spine motion data used to calculate stiffness through range	Low: 0.0 (0.1) Nm/%ROM Transition: 0.1 (0.1) Nm/%ROM High: 0.7 (0.3) Nm/%ROM %ROM = percent of ROM achieved in first trial on measurement rig
De Carvalho and Callaghan [4]	• 20 participants • 10 females and 10 males • Female (averages): ○ Age: 25.2 (3.2) years ○ Height: 1.70 (0.04) cm ○ Mass: 67.1 (7.6) kg ○ BMI: 23.1 (2.3) kg/m^2^ • Male (averages): ○ Age: 26.4 (3.5) years ○ Height: 1.79 (0.10) m ○ Mass: 82.7 (15.8) kg ○ BMI: 25.7 (3.4) kg/m^2^	• Assessing flexion in side-lying • Participant lying on side on two separate platforms • Legs fixed to static platform and torso on ‘frictionless’ moveable platform • Lumbar spine movements measured using two accelerometers on L1 and S1 spinous processes respectively •EMG electrodes placed on erector spinae muscle bellies bilaterally at T9 and L3 level • Passivity considered EMG amplitude less than 5% of MVC • A participant’s torso was pulled using a perpendicular force to the line of the body applied to the top of the torso platform, measured by a force transducer attached to the pulling cable • Time synchronised force and lumbar spine motion data used to calculate stiffness through range	Males: Low: 2.42 (2.07) Nm/° Transition: 0.76 (0.17) Nm/° High: 1.90 (0.67) Nm/° Females: Low: 0.96 (0.68) Nm/° Transition: 0.51 (0.17) Nm/° High: 1.98 (1.65) Nm/° Male and female combined into quartiles (calculated for present review) 1st quartile: 1.69 Nm/° 2nd quartile: 0.64 Nm/° 3rd quartile: 0.64 Nm/° 4th quartile: 1.94 Nm/°
Fewster et al. [5]	• 24 participants • 12 females and 12 males • Age: 26.3 (3.7) • Height: 1.71 (0.08) m • Mass: 76.2 (12.3) kg	• Assessing flexion and extension in side-lying • Participant lying on side on two separate platforms • Legs (pelvis down) fixed to static platform and torso on ‘frictionless’ moveable platform • Lumbar spine movements were measured using a optoelectronic motion capture system with lumbar spine defined by sensors on L1 and sacrum • EMG electrodes placed bilaterally on erector spinae muscle bellies at T9 and L3 level, the rectus abdominis and external obliques • Passivity considered EMG amplitude less than or equal to 5% of MVC • A participant’s torso was pulled using a perpendicular force to the line of the body applied to the top of the torso platform, measured by a force transducer attached to the pulling cable • Time synchronised force and lumbar spine motion data used to calculate stiffness through range	Male: Low: 0.33 (0.10) Nm/%Flexion Transition: 0.44 (0.23) Nm/%Flexion High: 1.42 (0.17) Nm/%Flexion Female: Low: 0.50 (0.13) Nm/%Flexion Transition: 0.38 (0.31) Nm/%Flexion High: 1.56 (0.33) Nm/%Flexion Average across genders: Low: 0.42 Nm/%Flexion Transition: 0.41 Nm/%Flexion High: 1.49 Nm/%Flexion %Flexion = percent of maximum flexion ROM achieved
Gruevski and Callaghan [7]	• 34 participants • 17 mature and 17 young participants • Mature participants average age: 63.7 (3.9) years • Young group baseline average data: ○ Age: 25.8 (5.0) ○ Female: ▪ Height: 1.62 (0.07) m ▪ BMI: 22.8 (1.8) kg/m^2^ ▪ Waist circumference: 72.0 (8.2) cm ▪ Physical activity: 2309 (566) MET-minutes/week ○ Male: ▪ Height: 1.78 (0.07) cm ▪ BMI: 26.1 (4.1) kg/m^2^ ▪ Waist circumference: 88.3 (10.5) cm ▪ Physical activity: 1858 (578) MET-minutes/week	• Assessing flexion and extension in side-lying • Participant lying on side on two separate platforms • Legs (pelvis down) fixed to static platform and torso on ‘frictionless’ moveable platform • Lumbar spine movements were measured using a four-camera optoelectronic motion capture system with lumbar spine defined by sensors on L1 and sacrum • EMG electrodes placed bilaterally on erector spinae muscle bellies at T9 and L3 level, the rectus abdominis and external obliques • Passivity considered EMG amplitude less than or equal to 5% of MVC • A participant’s torso was pulled using a perpendicular force to the line of the body applied to the top of the torso platform, measured by a force transducer attached to the pulling cable • Time synchronised force and lumbar spine motion data used to calculate stiffness through range	Passive stiffness at 40% total flexion: 0.10 (0.05) Nm/° Interpreted to represent second quartile stiffness for present review
Lee and McGill [9]	• 24 participants • All male • Baseline average data: ○ Age: 22.9 (2.7) years ○ Height: 1.79 (0.06) m ○ Mass: 77.5 (10.8) kg	• Assessed flexion/extension in side-lying • Legs (pelvis down) fixed to static platform and torso on ‘frictionless’ moveable platform • Three-dimensional lumbar spine movements measured using electromagnetic tracking system with the source placed on the sacrum and the sensor over T12 • EMG electrodes placed on rectus abdominis, external obliques, internal obliques, latissimus dorsi, upper erector spinae and lower erector spinae • Passivity considered EMG amplitude less than 5% of MVC • A participant’s torso was pulled using a perpendicular force to the line of the body applied to the top of the torso platform, measured by a force transducer	Results divided into two groups, one inexperienced in core strengthening training and one experienced in it Flexion: Inexperienced group: ROM achieved at varying percentages of total applied moment: 50%: 19.7 (8.1)° 65%: 22.1 (10.4)° 80%: 25.9 (12.7)° 90%: 31.8 (11.4)° 95%: 34.2 (11.2)° 100%: 35.6 (11.2)° Experienced group: ROM achieved at varying percentages of total applied moment: 50%: 19.5 (8.0)° 65%: 23.3 (8.5)° 80%: 27.6 (9.7)° 90%: 28.4 (11.7)° 95%: 30.1 (11.1)° 100%: 31.1 (10.9)° Extension: Inexperienced group: ROM achieved at varying percentages of total applied moment: 50%: 12.4 (8.2)° 65%: 16.0 (9.8)° 80%: 20.0 (11.4)° 90%: 21.0 (11.8)° 95%: 23.9 (11.2)° 100%: 25.6 (11.1)° Experienced group: ROM achieved at varying percentages of total applied moment: 50%: 15.9 (8.0)° 65%: 19.6 (8.4)° 80%: 18.6 (9.1)° 90%: 25.5 (7.7)° 95%: 28.7 (8.0)° 100%: 30.5 (8.4)°
McGill et al. [10]	• 37 participants • 15 females and 22 males • Female (average): ○ Age: 20.8 (1.8) years ○ Mass: 62.3 (8.8) kg ○ Height: 1.65 (0.05) m • Male (averages): ○ Age: 21.1 (1.2) years ○ Mass: 74.9 (7.6) kg ○ Height: 1.77 (0.06) m	• Assessed flexion/extension in side-lying • Legs (pelvis down) fixed to static platform and torso on ‘frictionless’ moveable platform • Three-dimensional lumbar spine movements measured using electromagnetic tracking system with the source placed over the pelvis and the sensor over the xiphoid process • EMG electrodes placed on the spine extensors at the L3 level and abdominal obliques • EMG signal presented as audio feedback and passivity considered when myoelectric silence was achieved throughout • A participant’s torso was pulled using a perpendicular force to the line of the body applied to the top of the torso platform, measured by a force transducer • Torque and rotational displacement data were time synchronised and were plotted against each other • Exponential curves were fit, and the derivative equation of this curve was used to calculate the stiffness	Passive stiffness (Nm/°) associated with degree through ROM: Flexion: 2 - 0.29 4 - 0.36 6 - 0.45 8 - 0.56 10 - 0.69 12 - 0.86 14 - 1.08 16 - 1.34 Extension: 2 - 0.17 4 - 0.20 6 - 0.23 8 - 0.28 10 - 0.32 12 - 0.38 14 - 0.45 16 - 0.53 18 - 0.62 20 - 0.73 22 - 0.86 Quartiles calculated for present review for flexion: 1st: 0.36 Nm/° 2nd: 0.76 Nm/° 3rd: 1.46 Nm/° 4th: 2.78 Nm/°
Shojaei et al. [15]	• 20 participants aged between 18 and 30 years old • 10 males and 10 females • Female (averages): ○ Height: 1.65 (0.05) cm ○ Mass: 67.1 (7.0) kg ○ BMI 24.7 (3.8) kg/m^2^ • Male (averages): ○ Height: 1.78 (0.06) m ○ Mass: 78.9 (12.0) kg ○ BMI 24.8 (3.5) kg/m^2^	• Assessed flexion in standing • Participants stood in a rigid metal hinged frame with their torso fixed in an upright position using a harness connected to a rigid rod • The participants legs and pelvis were constrained to the bottom half of the frame • The frame was adjusted such that the frame’s hinge was aligned with the participant’s S1 spinal level • EMG electrodes placed bilaterally on erector spinae muscle bellies at L3 and L5 level, the rectus abdominis and external obliques • EMG signal was monitored throughout to ensure their level of activity did not change • The participants’ legs were lifted forward/upwards by the frame, and the angle through which they moved was measured using a protractor attached to the leg of the frame • An inline load cell on the harness connecting rod assembly was used to measure the trunks response to the lumbar flexion • Stiffness was calculated from the change in moment and change of range • Participants only taken to 70% of total available passive range	Mean stiffness for quartiles of 70% total range 1st: 0.84 (0.53) Nm/° 2nd: 0.99 (0.66) Nm/° 3rd: 0.95 (0.81) Nm/° 4th: 1.60 (0.70) Nm/° 0–10% of ROM: 0.87 (0.50) Nm/° Quartiles of stiffness for total available range calculated from this study and converted to Nm/° for the current review: 1st: 0.92 2nd: 0.95 3rd: 1.60
Tennant et al. [17]	• 71 participants • 37 females and 34 males • Baseline average data for all participants: ○ Age: 26.2 (7.1) years ○ Height: 1.71 (0.82) m ○ Mass: 70.6 (11.3) kg ○ BMI: 24.0 (2.6) kg/m^2^ • Female (average) ○ Age: 27.4 (8.4) years ○ Height: 1.66 (7.22) m ○ Mass: 64.4 (9.2) kg ○ BMI: 23.2 (2.4) kg/m^2^ • Male (averages) ○ Age: 25.0 (5.1) years ○ Height: 1.76 (6.20) m ○ Mass: 77.4 (9.4) kg ○ BMI: 25.0 (2.5) kg/m^2^	• Flexion measured in side-lying • Participants’ low body (pelvis and distal) was constrained on an immobile platform with the upper body fixed to a ‘frictionless’ mobile cradle • Range of lumbar movement was measured by a digital camera recording the relative motion of reflective markers placed over the T12 and S2 spinous processes • EMG electrode placed on lumbar erector spinae at L3 level on nondominant side • Passivity assumed if muscle activity was less than 2% of the peak of two trials • The participants were drawn into maximum lumbar flexion via a rigid bar with a uniaxial load cell attached to it • The moment and angle data were time synchronised and plotted with the curves being approximated into three linear regions, the gradients of which represented the stiffness at these different stages	Low: 0.2 (0.1) Nm/° High: 2.26 (1.25) Nm/° For the current review, the low zone is considered to represent the first quartile and the high zone is considered to represent the fourth quartile
Toosizadeh et al. [18]	• 10 participants • 5 females and 5 males • Female (averages): ○ Age: 23.8 (2.6) years ○ Height: 1.64 (0.04) m ○ Mass 57.9 (5.1) kg • Male (averages): ○ Age: 24.4 (4.2) years ○ Height: 1.80 (0.07) m ○ Mass: 71.0 (7.3) kg	• Flexion measured in standing • Participants stood in a rigid metal hinged frame with their torso fixed in an upright position using a harness connected to a rigid rod • The participants legs and pelvis were constrained to the bottom half of the frame • The frame was adjusted such that the frame’s hinge was aligned with the L5/S1 joint of the participant • EMG electrodes were placed on longissimus at the L3 level and rectus abdominis at the level of the umbilicus • EMG signal was monitored to minimize voluntary muscle activation throughout; however, no specific passive threshold was specified • The participants’ legs were lifted forward/upwards by the frame, and the angle through which they moved was measured using inertial measurement units placed over the spinous processes of T12 and S1 • An inline load cell on the harness connecting rod assembly was used to measure the trunk’s response to the lumbar flexion • Stiffness was calculated using four different models: standard linear solid, Prony series, Schapery’s theory and the modified superposition method	Stiffness (Nm/°) for specific percentages of the flexion relaxation angle calculated by two different means Prony series: 30%: 0.07 (0.14) 40%: 0.19 (0.12) 60%: 0.19 (0.23) 80%: 0.24 (0.28) 100%: 0.23 (0.25) Schapery’s theory: 30%: 0.15 (0.01) 40%: 0.15 (0.01) 60%: 0.26 (0.01) 80%: 0.23 (0.01) 100%: 0.54 (0.01)
Voinier et al. [20]	• 31 participants • Control group baseline average data ○ Age: 30.00 (9.32) years ○ Female: 47% ○ Height: 1.75 (0.10) m ○ Mass: 74.0 (9.9) kg ○ BMI 24.1 (2.0) kg/m^2^	• Assessed flexion/extension in side-lying • Legs (pelvis and distal) fixed to static platform and torso on ‘frictionless’ moveable platform • Angular displacement was measured using inertial measurement units, one placed on the pelvis and one on the torso • A load cell was attached to the moveable platform via a ball and socket joint with an inertial measurement unit attached to it to monitor the angle of pull applied • EMG electrodes were placed over the external obliques and lumbar erector spinae muscles • Baseline EMG levels were established once a subject has been placed within the apparatus • Non-passive trials were identified if the EMG level surpassed the level of two standard deviations of this baseline level for more than 10% of the trial • Participants were pulled into the respective movement to be measured while the displacement and force were recorded • The time synchronised torque and displacement data were fit to a double sigmoid curve, the derivative of which described the inverse of stiffness	Mean neutral zone stiffness (Nm/°) in flexion/extension: 0.18 (0.06) Considered to represent the first quartile for the current review

Where possible, results were converted to Newton metres per degree. Participant data was only presented for those participants relevant to the current review. Stiffness values calculated specifically for this review are clearly identified in the stiffness results column. Numbers in brackets represent standard deviations.

*m* metres, *kg* kilograms, *Nm* Newton metre, *ROM* range of movement, *T9* ninth thoracic vertebrae, *T12* twelfth thoracic vertebrae, *L1* first lumbar vertebrae, *L3* third lumbar vertebrae, *L5* fifth lumbar vertebrae, *S1* first sacral vertebrae, *S2* second sacral vertebrae, *MET* metabolic equivalent, *BMI* body mass index.

**Table 3:** Data extraction table for studies investigating lateral bending

Author and reference	Participants	Methods for stiffness measurements	Results
Gombatto et al. [6]	• 50 participants • 31 with lower back pain and 19 without • Baseline average data for those without lower back pain: ○ 9 females and 10 males ○ Age: 30.3 (8.5) years ○ Height: 1.69 (0.10) m ○ Mass: 70.2 (15.1) kg ○ BMI: 24.2 (3.0) kg/m^2^ ○ Baecke Score: 8.9 (1.0)	• Lateral bending assessed in prone • Pelvis and distal secured to a static table • Participant’s trunk secured to a ‘frictionless’ moveable cradle • EMG electrodes were placed over the external obliques and lumbar erector spinae bilaterally • A passive trial was considered to be any trial where the activity of the muscles opposing the movement did not exceed two percent of the MVC for 0.3 s or more at any point during the trial • A participant’s torso was pulled using a perpendicular force to the line of the body applied to the top of the cradle, measured by a force transducer attached to the pulling cable • A six-camera motion capture system was used to measure the ROM of the lumbar spine with markers located superficial to the first lumbar vertebra and a triad of markers over the second sacral spinous process • Time synchronised force and lumbar spine motion data used to calculate stiffness through range	Stiffness (Nm/°) values for each quartile 0–25% angle Left: Test 1: 0.23 (0.17) Test 2: 0.25 (0.16) Test 3: 0.26 (0.17) Right: Test 1: 0.26 (0.19) Test 2: 0.29 (0.21) Test 3: 0.31 (0.18) 25–50% angle Left: Test 1: 0.61 (0.29) Test 2: 0.63 (0.28) Test 3: 0.64 (0.31) Right: Test 1: 0.64 (0.36) Test 2: 0.76 (0.49) Test 3: 0.75 (0.39) 50–75% angle Left: Test 1: 1.71 (0.61) Test 2: 1.74 (0.78) Test 3: 1.63 (0.62) Right: Test 1: 1.74 (0.78) Test 2: 2.03 (1.24) Test 3: 1.91 (0.95) 75–100% angle Left: Test 1: 5.19 (2.49) Test 2: 5.25 (3.38) Test 3: 4.79 (2.75) Right: Test 1: 5.14 (2.91) Test 2: 5.78 (3.54) Test 3: 5.07 (2.72) Values averaged across the three tests to establish quartiles for the current study: 1st quartile: 0.27 Nm/° 2nd quartile: 0.67 Nm/° 3rd quartile: 1.79 Nm/° 4th quartile: 5.20 Nm/°
Lee and McGill [9]	• 24 participants • All male • Baseline average data: ○ Age: 22.9 (2.7) years ○ Height: 1.79 (0.06) m ○ Mass: 77.5 (10.8) kg	• Lateral bending in supine lying • Legs (pelvis and distal) fixed to static platform and torso on ‘frictionless’ moveable platform • Three-dimensional lumbar spine movements measured using electromagnetic tracking system with the source placed over the lower abdomen at a level slightly below the ASIS and the sensor over the xiphoid process • EMG electrodes placed on rectus abdominis, external obliques, internal obliques, latissimus dorsi, upper erector spinae and lower erector spinae • Passivity considered EMG amplitude less than 5% of MVC • A participant’s torso was pulled using a perpendicular force to the line of the body applied to the top of the torso platform, measured by a force transducer • Stiffness was not directly reported; however, normalised (to the maximum applied moment) passive moment was calculated and reported alongside range of movement time normalised	Results divided into two groups, one inexperienced in core strengthening training and one experienced in it Right bend: Inexperienced group: ROM achieved at varying percentages of total applied moment: 50%: 13.9 (4.8)° 65%: 17.0 (4.3)° 80%: 18.8 (7.7)° 90%: 22.1 (6.3)° 95%: 23.6 (5.7)° 100%: 24.5 (5.5)° Experienced group: ROM achieved at varying percentages of total applied moment: 50%: 14.1 (11.0)° 65%: 13.9 (10.3)° 80%: 17.6 (10.5)° 90%: 20.5 (10.5)° 95%: 21.7 (10.6)° 100%: 22.5 (10.6)° Left bend: Inexperienced group: ROM achieved at varying percentages of total applied moment: 50%: 10.7 (7.2)° 65%: 13.6 (6.4)° 80%: 17.3 (8.9)° 90%: 21.6 (9.4)° 95%: 23.6 (9.8)° 100%: 24.7 (10.0)° Experienced group: ROM achieved at varying percentages of total applied moment: 50%: 13.6 (7.2)° 65%: 15.7 (6.9)° 80%: 16.9 (8.1)° 90%: 19.5 (8.2)° 95%: 21.6 (8.1)° 100%: 22.8 (8.3)°
McGill et al. [10]	• 37 participants • 15 females and 22 males • Female (average): ○ Age: 20.8 (1.8) years ○ Height: 1.65 (0.05) m ○ Mass: 62.3 (8.8) kg • Male (averages): ○ Age: 21.1 (1.2) years ○ Height: 1.77 (0.06) m ○ Mass: 74.9 (7.6) kg	• Lateral bending in supine lying • Legs (pelvis and distal) fixed to static platform and torso on ‘frictionless’ moveable platform • Three-dimensional lumbar spine movements measured using electromagnetic tracking system with the source placed over the pelvis and the sensor over the xiphoid process • EMG electrodes placed on the spine extensors at the L3 level and abdominal obliques • EMG signal presented as audio feedback and passivity considered when myoelectric silence was achieved throughout • A participant’s torso was pulled using a perpendicular force to the line of the body applied to the top of the torso platform, measured by a force transducer • Torque and rotational displacement data were time synchronised and were plotted against each other • Exponential curves were fitted, and the derivative equation of this curve was used to calculate the stiffness	Passive stiffness (Nm/°) associated with degree through ROM of right lateral bend: 2 - 0.32 4 - 0.40 6 - 0.49 8 - 0.61 10 - 0.75 12 - 0.93 14 - 1.14 16 - 1.41 18 - 1.75 20 - 2.16 Quartiles calculated for present study: 1st: 0.45 Nm/° 2nd: 1.05 Nm/° 3rd: 2.35 Nm/° 4th: 4.79 Nm/°
Voinier et al. [20]	• 31 participants • Control group baseline average data ○ Age: 30.00 (9.32) years ○ Female: 47% ○ Height: 1.75 (0.10) m ○ Mass: 74.0 (9.9) kg ○ BMI 24.1 (2.0) kg/m^2^	• Lateral bending in supine lying • Legs (pelvis and distal) fixed to static platform and torso on ‘frictionless’ moveable platform • Angular displacement was measured using inertial measurement units, one placed on the pelvis and one on the torso • A participant’s torso was pulled using a perpendicular force to the line of the body applied to the top of the torso platform, measured by a force transducer • A load cell was attached to the moveable platform via a ball and socket joint with an inertial measurement unit attached to it to monitor the angle of pull applied • EMG electrodes placed over the external obliques and lumbar erector spinae muscles • Baseline EMG levels were established once a subject has been placed within the apparatus • Non-passive trials were identified if the EMG level surpassed the level of two standard deviations of this baseline level for more than 10% of the trial • Participants were pulled into the respective movement to be measured while the displacement and force were recorded • The time synchronised torque and displacement data were fit to a double sigmoid curve, the derivative of which described the inverse of stiffness	Mean neutral zone stiffness (Nm/°) in lateral bending: 0.20 (0.06) Considered to represent the first quartile for the current study

Where possible, results were converted to Newton metres per degree. Participant data was only presented for those participants relevant to the current review. Stiffness values calculated specifically for this review are clearly identified in the stiffness results column. Numbers in brackets represent standard deviations.

*L3* third lumbar vertebrae, *m* metres, *kg* kilograms, *Nm* Newton metre, *ROM* range of movement, *ASIS* anterior superior iliac spine, *BMI* body mass index.

**Table 4:** Data extraction table for studies investigating axial rotation

Author and reference	Participants	Methods for stiffness measurements	Results
Kosmopoulos et al. [8]	• 18 participants • 9 females and 9 males • Female (averages): ○ Age: 20.8 (2.0) years ○ Height: 1.63 (0.05) m ○ Mass: 65.5 (12.1) kg ○ BMI: 24.7 (4.6) kg/m^2^ • Male (averages): ○ Age: 21.1 (2.6) years ○ Height: 1.74 (0.08) m ○ Mass: 79.4 (18.1) kg ○ BMI 26.5 (5.3) kg/m^2^	• Assessed axial rotation in sitting • The lower half of participants bodies (hips and distal) were fixed down to prevent any movement • A shoulder harness was firmly fixed to participants and linked to an overhead circular pulley system • No description of how the authors ensured a trial was passive has been provided • Weights of known quantity were added to this pulley to impart a rotational moment on the thoracolumbar spine • The angle of rotation was measured using a high precision potentiometer • The stiffness was calculated as the gradient of the line of best fit when the moment and angle of rotation were plotted together	Average left rotational stiffness (all): 0.30 (0.09) Nm/° Average right rotational stiffness (all): 0.35 (0.10) Nm/° Male average rotational stiffness Left: 0.34 (0.09) Nm/° Right: 0.39 (0.10) Nm/° Female average rotational stiffness: Left: 0.27 (0.07) Nm/° Right: 0.31 (0.08) Nm/° Left and right rotational stiffnesses averaged and interpreted to represent quartiles one and two: 0.33 Nm/°
Lee and McGill [9]	• 24 participants • All male • Baseline average data: ○ Age: 22.9 (2.7) years ○ Height: 1.79 (0.06) m ○ Mass: 77.5 (10.8) kg	• Axial rotation in standing • Participants stood on a ‘frictionless’ rotatable platform • Participants’ torsos were fixed to a harness strapped to a vertical post • The method for measuring the three-dimensional lumbar spine movements was not reported for axial rotation but was performed using an electromagnetic transducer • EMG electrodes placed on rectus abdominis, external obliques, internal obliques, latissimus dorsi, upper erector spinae and lower erector spinae • Passivity considered EMG amplitude less than 5% of MVC • The rotating platform on which participants stood was rotated using a cable attached to the platform, and the force with which this was pulled was measured using a force transducer • Stiffness was not directly reported; however, normalised (to the maximum applied moment) passive moment was calculated and reported alongside ROM time normalised	Results divided into two groups, one inexperienced in core strengthening training and one experienced in it Right twist: Inexperienced group: ROM achieved at varying percentages of total applied moment: 50%: 5.2 (3.0)° 65%: 7.4 (3.2)° 80%: 9.1 (4.2)° 90%: 10.5 (4.7)° 95%: 11.1 (5.0)° 100%: 11.5 (5.1)° Experienced group: ROM achieved at varying percentages of total applied moment: 50%: 7.5 (3.5)° 65%: 9.0 (4.0)° 80%: 12.3 (4.3)° 90%: 14.2 (4.6)° 95%: 15.0 (4.7)° 100%: 15.5 (4.8)° Left Twist: Inexperienced group: ROM achieved at varying percentages of total applied moment: 50%: 6.5 (2.4)° 65%: 8.1 (3.4)° 80%: 10.8 (4.5)° 90%: 12.3 (5.3)° 95%: 13.0 (5.6)° 100%: 13.4 (5.9)° Experienced group: ROM achieved at varying percentages of total applied moment: 50%: 6.9 (2.3)° 65%: 9.4 (2.9)° 80%: 13.5 (3.3)° 90%: 15.8 (3.8)° 95%: 16.9 (4.1)° 100%: 17.5 (4.3)°
McGill et al. [10]	• 37 participants • 15 females and 22 males • Female (average): ○ Age: 20.8 (1.8) years ○ Height: 1.65 (0.05) m ○ Mass: 62.3 (8.8) kg • Male (averages): ○ Age: 21.1 (1.2) years ○ Height: 1.77 (0.06) m ○ Mass: 74.9 (7.6) kg	• Axial rotation in standing • Participants stood on a ‘frictionless’ rotatable platform • Their torsos were fixed to a harness strapped to a vertical post • Three-dimensional lumbar spine movements measured using electromagnetic tracking system with the source placed over the pelvis and the sensor over the xiphoid process • EMG electrodes placed on the spine extensors at the L3 level and abdominal obliques • EMG signal presented as audio feedback and passivity considered when myoelectric silence was achieved throughout • The rotating platform on which participants stood was rotated using a cable attached to the platform, and the force with which this was pulled was measured using a force transducer • Torque and rotational displacement data were time synchronised and were plotted against each other • Exponential curves were fitted, and the derivative equation of this curve was used to calculate the stiffness	Passive stiffness (Nm/°) associated with degree through ROM of clockwise twist: 2 - 0.13 4 - 0.15 6 - 0.17 8 - 0.20 10 - 0.23 12 - 0.26 14 - 0.31 16 - 0.36 18 - 0.41 20 - 0.48 22 - 0.56 24 - 0.64 Quartiles calculated for present study: 1st: 0.17 Nm/° 2nd: 0.35 Nm/° 3rd: 0.71 Nm/° 4th: 1.33 Nm/°
Tsung et al. [19]	• 20 participants • 6 females and 14 males • Baseline average data: ○ Age: 28.0 (6.3) years ○ Height: 1.71 (0.08) m ○ Mass: 62.5 (11.6) kg	• Axial rotation measured in side-lying • Participants were placed in side-lying with hips and knees flexed on a manipulation plinth with two sections, a sensing section on which the lower half of the body was place, and a supporting section on which the upper half of the body was situated • A force plate capable of tri-axial force measurements was situated under the sensing section • The spinal section to which a mobilisation was to be applied was aligned to the junction between the two sections of the plinth • Lumbar spine motion was measured using an electro-magnetic motion tracking system with sensors being placed over the spinous process of L1 and the sacrum • No description of how the authors ensured a trial was passive has been provided • Mobilisations of different grades (I-IV) were applied and stiffness was calculated from the ratio of twisting moment amplitude compared to amplitude of axial rotation	Mean stiffness for: Grade I = 3.7 ± 0.3 Nm/° Grade II = 3.8 ± 0.9 Nm/° Grade III = 5.4 ± 0.8 Nm/° Grade IV = 7.1 ± 0.9 Nm/°
Voinier et al. [20]	• 31 participants • Control group baseline average data ○ Age: 30.0 (9.3) years ○ Female: 47% ○ Height: 1.75 (0.10) m ○ Mass: 74.0 (9.9) kg ○ BMI 24.1 (2.0) kg/m^2^	• Axial rotation in kneeling • Participants knelt in an ergonomic chair mounted on a rotating platform • The torso was fixed in position by adjustable underarm supports, and the lower extremities were constrained to the chair • Angular displacement was measured using inertial measurement units, one placed on the pelvis and one on the torso • A load cell was attached tangentially to the rotating platform to measure the force with which participants were pulled • EMG electrodes placed over the external obliques and lumbar erector spinae muscles • Baseline EMG levels were established once a subject has been placed within the apparatus • Non-passive trials were identified if the EMG level surpassed the level of two standard deviations of this baseline level for more than 10% of the trial • Participants were pulled into the respective movement to be measured while the displacement and force were recorded • The time synchronised torque and displacement data were fit to a double sigmoid curve, the derivative of which described the inverse of stiffness	Mean neutral zone stiffness (Nm/°) in flexion/extension: 0.16 (0.08) Considered to represent the 1st quartile for the current study

Where possible, results were converted to Newton metres per degree. Participant data was only presented for those participants relevant to the current review. Stiffness values calculated specifically for this review are clearly identified in the stiffness results column. Numbers in brackets represent standard deviations.

*m* metres, *kg* kilograms, *Nm* Newton metre, *ROM* range of movement, *L1* first lumbar vertebrae, *L3* third lumbar vertebrae, *BMI* body mass index.

The way these values were calculated varied depending on the methods by which they were reported. For studies that found a linear relationship between moment and ROM, the stiffness was considered to be constant throughout all four quartiles.

For studies that presented single point values of stiffness for multiple points throughout the range, the gradient of the line that would link the two end points of a quartile, on a moment against ROM graph, was calculated. An example of how this is represented is provided in Fig. 2.

For studies that presented their results as low (or neutral), transition and high stiffness zones, the results for these zones were considered to be comparative to certain quartiles. The low zone was considered to represent the first quartile in all cases, and the high zone was considered to be representative of the fourth quartile. How the second and third quartiles were represented depended on the study in question. Some were interpreted such that the low zone also represented the second quartile, whereas in others, the transition zone was considered to represent the second and third quartiles. The decision as to which quartiles these zones were taken to represent was based, where possible, either on the description of how they established these zones or on their graphical representations of their results and through consensus by authors.

Some studies artificially limited the ROM their participants were exposed to, for instance, Shojaei et al. [15] who only took their participants to 70% of their maximum ROM. For studies such as this, where their results were presented as quartiles, but of this reduced ROM, their results were converted to the ranges they represented for a ROM of 100%. For instance, the first quartile (0–25%) of 70% ROM represents the stiffness for the range of 0–17.5% ROM for the maximum achievable ROM. Therefore, 7.5% of the stiffness for quartile one of the maximum ROM is represented by the second quartile stiffness of the 70% range. Knowing this, a weighted mean stiffness was calculated for each quartile.

Once the quartiles were established for all the studies where this was possible, studies investigating the same plane of movement were compared and weighted stiffness means were established for each quartile. This weighting was provided by the number of participants in each study whose data was considered relevant as described above. As well as a weighted mean, a standard deviation for each quartile was calculated from the mean stiffness values provided by/calculated from each study for each quartile. From these standard deviations, the standard error of the mean was calculated for each quartile and, using this, the confidence intervals for 90%, 95% and 99% of the observations were established.

## Results

Included within this study were thirteen articles, ten of which investigated flexion/extension [2, 4, 5, 7, 9, 10, 15, 17, 18, 20], four that studied lateral bending [6, 9, 10, 20] and five that examined axial rotation [8–10, 19, 20]. These studies were published between 1994 and 2021 with a total of 354 participants studied who were relevant to this review. The average ages of study participants ranged from 21.0 to 30.3 years. A data extraction table for the included studies split into the respective movements investigated can be seen in Tables 2, 3 and 4.

### Flexion/extension

**Fig. 3:**
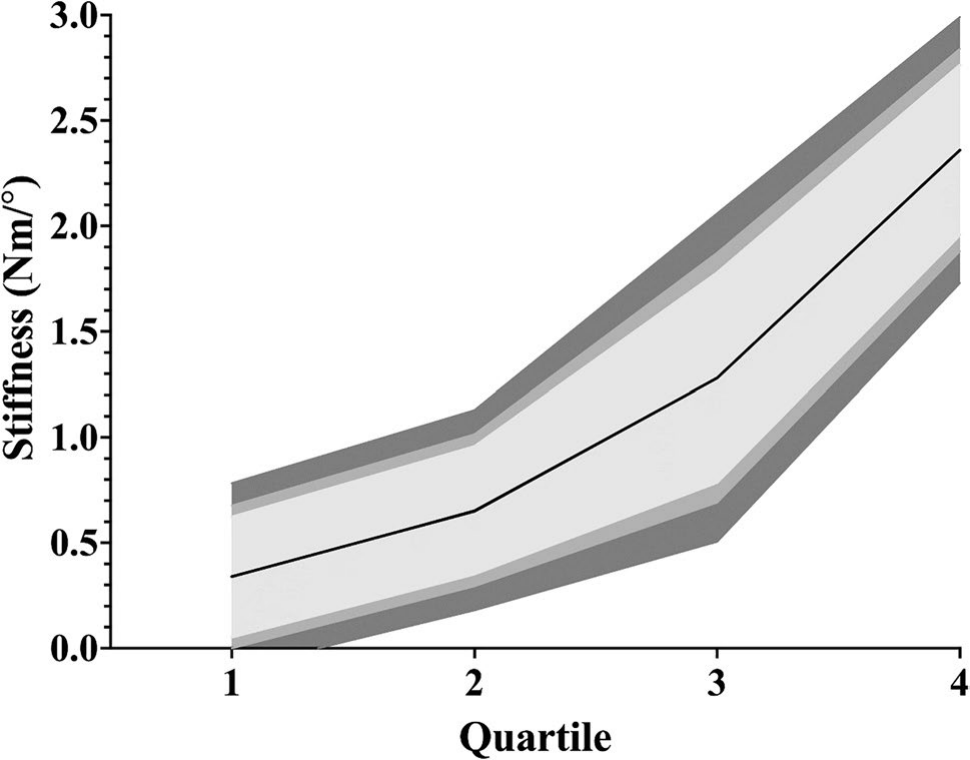
Flexion synthesised weighted mean stiffnesses for each quartile with confidence intervals. The area demarcated by lightest grey represents 90% confidence interval. The area encompassing the two lightest greys represents the 95% confidence interval. The three grey regions combined represent the 99% confidence interval. Nm/°, Newton metres per degree

Within the ten articles (Table 2) that studied passive flexion/extension stiffness, eight conducted their measurements in side-lying [2, 4, 5, 7, 9, 10, 17, 20] and two performed theirs in standing [15, 18]. Six of these [4, 7, 10, 15, 17, 20] presented their values in a format suitable for comparison with the others in this context. The synthesised weighted mean stiffnesses and confidence intervals for the quartiles can be seen in Table 5 and graphically represented in Fig. 3.

**Table 5:** Synthesised weighted mean stiffness and 95% confidence interval values for flexion

Quartile	Weighted mean stiffness (Nm/°)	95% confidence interval (Nm/°)
1	0.34	0.00–0.68
2	0.65	0.29–1.02
3	1.28	0.69–1.87
4	2.36	1.88–2.84

*Nm/°* Newton metres per degree.

### Lateral bending

**Fig. 4:**
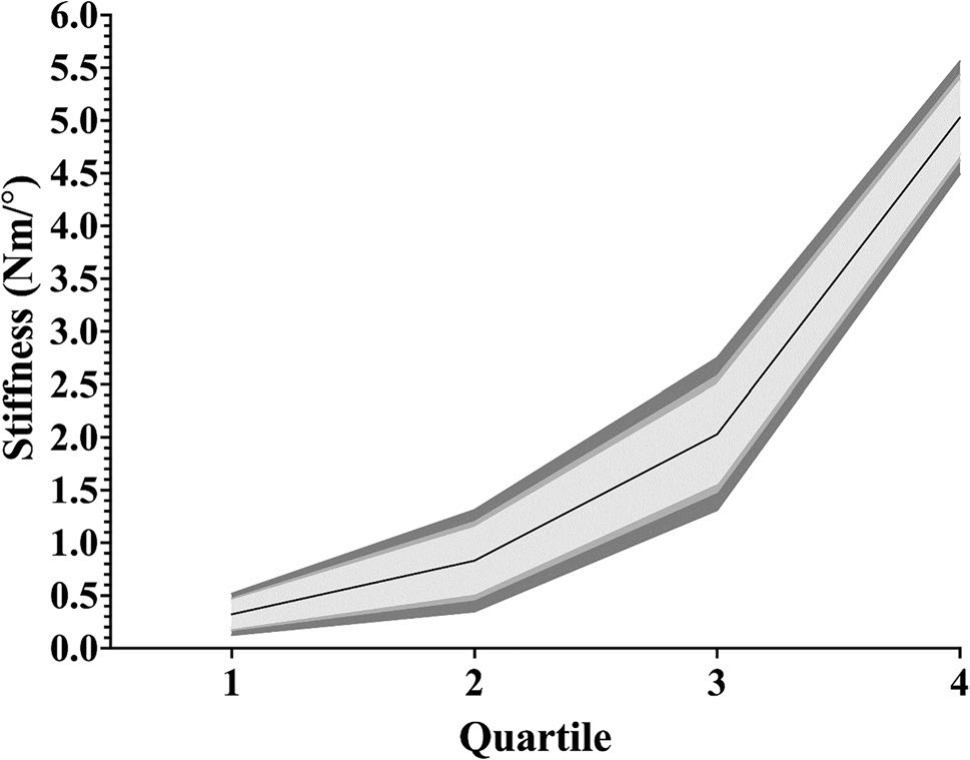
Lateral bending synthesised weighted mean stiffnesses for each quartile with confidence intervals. The area demarcated by lightest grey represents 90% confidence interval. The area encompassing the two lightest greys represents the 95% confidence interval. The three grey regions combined represent the 99% confidence interval. Nm/°, Newton metres per degree

Included in the four studies (Table 3) that investigated passive stiffness in lateral bending, three investigated it in supine lying [9, 10, 20] and one in prone lying [6]. Three of these studies [6, 10, 20] included numerical results suitable to be used in quantitative synthesis in this context. The synthesised weighted mean stiffnesses and confidence intervals for the quartiles can be seen in Table 6 and graphically displayed in Fig. 4.

**Table 6:** Synthesised weighted mean stiffness and 95% confidence interval values for lateral bending

Quartile	Weighted mean stiffness (Nm/°)	95% confidence interval (Nm/°)
1	0.32	0.17–0.47
2	0.83	0.46–1.20
3	2.03	1.48–2.58
4	5.03	4.62–5.43

*Nm/°* Newton metres per degree.

### Axial rotation

**Fig. 5:**
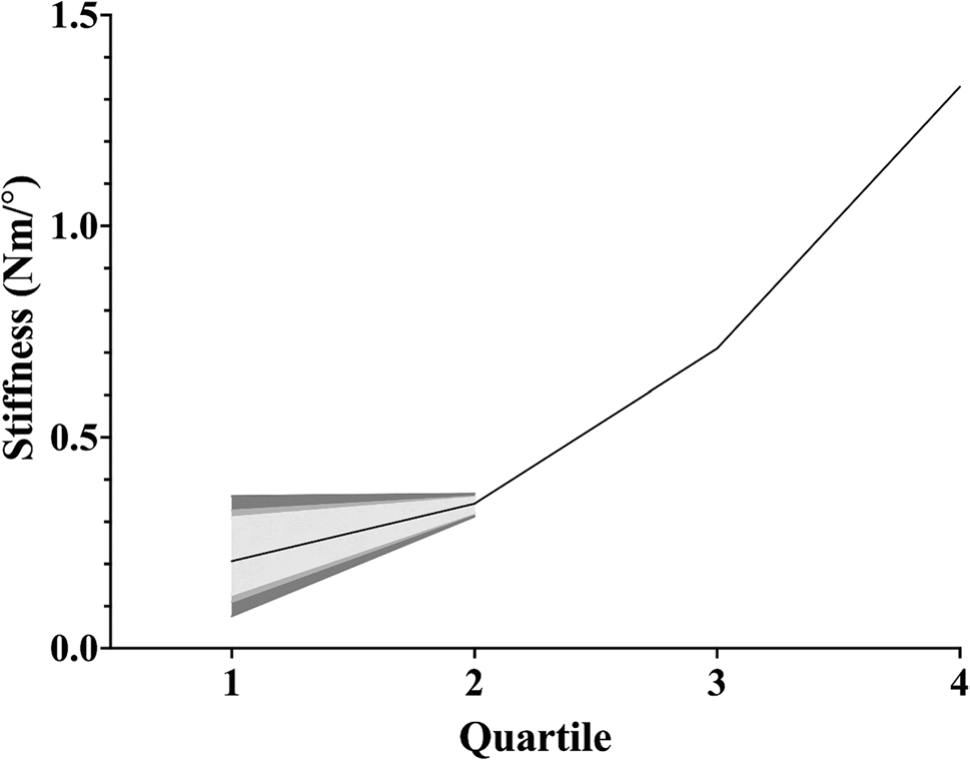
Axial rotation synthesised weighted stiffness values for axial rotation with confidence intervals. The area demarcated by lightest grey represents 90% confidence interval. The area encompassing the two lightest greys represents the 95% confidence interval. The three grey regions combined represents the 99% confidence interval. Confidence intervals for quartiles 3 and 4 were not calculable and therefore are not presented here. Nm/°, Newton metres per degree

Of the five studies (Table 4) investigating passive stiffness in axial rotation, two made their measurements in standing [9, 10], one in sitting [8], one sitting in an ergonomic chair [20] and one in side-lying [19]. Three of the studies provided numerical results for passive stiffness [8, 10, 20] in such a format that they could be used for quantitative synthesis in this context. The synthesised weighted mean stiffnesses and confidence intervals for the quartiles can be seen in Table 7 and graphically displayed in Fig. 5. Standard deviations and confidence intervals were not producible for the 3rd and 4th quartiles of axial rotation as only one article [10] using the relevant units investigated this portion of the motion.

**Table 7:** Synthesised weighted mean stiffness and 95% confidence interval values for axial rotation

Quartile	Weighted mean stiffness (Nm/°)	95% confidence interval (Nm/°)
1	0.21	0.10–0.32
2	0.34	0.32–0.36
3	0.71	N/A
4	1.33	N/A

*Nm/°* Newton metres per degree.

## Discussion

The primary aim of this study was to create a quantitative synthesis of values of stiffness for non-elderly adults for each of the three cardinal planes of movement. Despite the wide heterogeneity, this has been achieved and has yielded some interesting findings worthy of further discussion. Lateral bending was found to be the comparatively stiffest movement in vivo followed by flexion, extension and then axial rotation. This is in direct contradiction to that found in vitro for both cadaveric human and porcine spines [3, 22]. The reason behind the discrepancy is not immediately clear. One explanation may be the wide variety in the methodologies used to quantify in vivo stiffness. However, if this were the case, it might be expected that at least one study would contradict this pattern, yet this was not the case. Alternatively, it is possible that the distinct biomechanical difference between the spines studied in vitro and those in vivo could explain this difference. The removal of the muscles, rib cage and thoracolumbar fascia from the in vitro spine specimens may well influence the stiffness measurements. It would therefore suggest that the spinal musculature and fascia provide additional resistance to lateral bending. However, due to the paucity of studies investigating this topic, it is not possible to draw conclusions.

### Methodologies

There is a large amount of methodological variation in the studies included in this review. This may help to explain some of the variance in the mean estimates of stiffness. One key consideration is the method used to create a fixed segment and mobile segment. This was particularly evident in studies investigating flexion, extension and axial rotation. All but one of the studies included in the quantitative synthesis that investigated flexion used very similar, if not identical, methods with participants assessed in side-lying with their trunk on a ‘floating’ platform and their pelvis and legs fixed to a static platform. By moving the ‘floating’ platform by a known force and monitoring the relative motion of the lumbar spine, the stiffness of the spine in flexion was calculated. This contrasts with the methodology used by Shojaei et al. [15] who had participants stood upright in a motorised rig through which their hips (with legs kept straight) could be flexed while their torso was maintained in a fixed position. Here the angle of the platform which moved the legs was used to represent the ROM, and the moment was derived from the force applied to a pressure sensor located behind the torso, detecting changes in the amount of force with which the torso involuntarily attempted to extend as a result of the legs being raised.

This clear difference in methodology yielded consistently higher stiffness values being reported by Shojaei et al. [15] across the first three quartiles, most significantly the first quartile. The mean reported by Shojaei et al. [15] for the first quartile was over double that of the next nearest value [10] and nearly five times that of the lowest value [17]. This was consistent for the second and third quartiles as well with Shojaei et al.’s [15] stiffness value being ten times that of the lowest value found by another study for the second quartile [7]. However, for the latter two quartiles, Shojaei et al. [15] had relatively good agreement with McGill et al. [10]. This therefore suggests that the variability cannot be fully accounted for simply by how the studies have chosen to fixate their participants, especially when considering that there is still variability within the lateral bending studies which all fixated or moved the same parts of the body.

In addition to the fixation, the position in which participants were investigated is of importance. This is best demonstrated by the assessment of axial rotation where all three studies performed their measurements in three different ways, standing [10], sitting [8] and sitting in an ergonomic chair [20]. Each of these positions by their nature required the lumbar spine to adopt slightly different postures to maintain the torso in an upright position. It would therefore seem logical that there would be a large variance in the first quartile stiffnesses due to differences in pre-load of tissues in each of the postures. However, the exact opposite was found with the first quartile of axial rotation having a relatively low variance with it being the second lowest of all the quartiles in all the planes of movement studied. This therefore suggests the posture of the spine may affect stiffness in vivo in axial rotation very little in the early stages of ROM. It should be noted however that each of these postures is a weight-bearing posture. In the studies investigating flexion in the quantitative synthesis, Shojaei et al.’s study [15] was the only one to perform their assessment in standing, whereas the others all performed their stiffness assessments in side-lying. This may therefore further explain the relatively large reduction in mean stiffness when Shojaei et al.’s [15] results were removed from the comparison. This is in keeping with in vitro studies of the spine where stiffness characteristics increase in the spine with greater compressive loading [21]. Whether the spine is in a weight-bearing position is therefore a potentially important consideration when investigating the stiffness properties of the spine.

When considering lateral bending in the context of the previous paragraph, it may be reasonable to question whether the difference in assessing people in supine [10, 20] or prone [6] is what creates the variance in these studies. It is unlikely the stiffness properties themselves change drastically from prone to supine, but rather, the techniques used to observe these properties do. Due to the difference in positions between prone and supine, it would not be possible to use the same anatomical landmarks for sensors used to define the lumbar spine. McGill et al. [10] used the pelvis and the xiphoid process for their landmarks, whereas Gombatto et al. [6] utilised the first lumbar and second sacral spinous processes. This difference will inherently produce a difference in ROM observed and number of vertebral levels involved making it a likely source of variance between studies. Regarding axial rotation, the method adopted by McGill et al. [10] was different to that used by Kosmopoulos et al. [8]. McGill et al. [10] used electromagnetic sensors placed over the xiphoid process and the pelvis, whereas Kosmopoulos et al. [8] used a potentiometer built into their rig. Kosmopoulos et al. [8] had participants sat fixated in a chair with a shoulder harness. This shoulder harness was then rotated, and the angular displacement through which this harness rotated was measured using the potentiometer. The ROM recorded in their study therefore represents that of much of the thoracolumbar spine, rather than just the lumbar spine as in McGill et al.’s [10] study. This however seemed to have little effect on the stiffness values obtained with the first two quartiles of axial rotation yielding two of the lowest variances of all the movements analysed. However, there is insufficient data to conclude this for the later stages of the movement.

Additional differences were observed between studies from which variance in mean values may well be derived. Most studies, including McGill et al. [10], applied force to their participants in a continuous, gradually increasing manner. In contrast, Kosmopoulos et al. [8] applied known moments in a random order, recording the ROM achieved by applying each specific moment. Despite this fundamental difference in method, there was good agreement between the three studies investigating rotation included in the synthesis. It is not possible to conclude that the difference in spine length included and loading method have no great significance as McGill et al.’s study [10] was the only one investigating axial rotation that was interpreted to have taken participants to the end of their maximum available range. It is therefore highly possible that these differences have little effect in the first two quartiles but may produce much greater variance as the stiffness increases.

### Reporting

As with Kosmopoulos et al. [8], many of the studies included in this review have looked at certain portions of the range rather than investigating participants maximum passive ROM. This suits the needs of the individual studies but can hinder direct comparison in some cases. Especially when this is further confounded by the variety of ways, these results are interpreted by the authors. This was done in a variety of ways including but not limited to presenting the results as a continuous curve such as in McGill et al. [10], dividing the resultant curve into quartiles of a reduced range such as Shojaei et al. [15], dividing the curve into three portions: low, transition and high stiffness as first suggested by Punjabi [12] and being used by De Carvalho and Callaghan [4] among others or performing a linear regression on the results [8]. These differing techniques for categorising each study’s results will inherently produce variability between studies; however, where possible, these discrepancies have been mitigated in the present study’s comparison.

### Passivity

Despite all the studies included investigating passive stiffness, the way in which passivity was defined varied considerably between studies. The most common way to establish passivity was to monitor muscle activity relative to maximal voluntary contraction (MVC) using electromyography (EMG) [2, 4–7, 9]. Despite similarities in setup, a variety of thresholds at which muscles were deemed to be active were used, ranging from > 2% [6] to > 5% [2, 4, 5, 7, 9] MVC. Tennant et al. [17] followed a similar format but rather defined their threshold by the peak value experienced during two trials of sub-maximal contraction, setting their threshold for passivity as less than 2% of this peak. Voinier et al. [20] however took the opposite approach, recording a participant’s EMG activity when they were perfectly relaxed in their rig and considered a trial to be non-passive if their EMG level exceeded two standard deviations of this relaxed EMG value for more than 10% of a trial. Further to this, one study [10] projected their EMG signals audibly and defined their trial as passive if this was inaudible, whereas others did not report on their definition of passivity at all [8, 19]. It stands to reason that studies that allowed greater levels of muscular contraction should report greater values of stiffness. This however was not consistently the case with De Carvalho and Callaghan [4] (< 5% MVC threshold) reporting lower fourth quartile values than Tennant et al. [17] (passivity definition as discussed above). This may however be explained by the experiences of Lee and McGill [9] who provided the instruction to their participants to ‘feel completely relaxed like you are going to sleep’ and found that even though they set their threshold to 5% MVC, none of their participants exceeded 3% MVC during any of their trials. This therefore suggests that despite the thresholds varying between studies, it may be the instructions given to participants that hold more value. These instructions however were not consistently reported between studies; therefore, it is difficult to draw this conclusion with certainty.

### Participants

The final potential sources of variability to discuss are the participants themselves. As with any physical attribute, there is likely to be natural variability of this attribute within a group of people and this is likely to be true for passive spinal stiffness. However, it is important to recognise where the selection of participants for certain studies in this review may exacerbate this natural variability. For instance, most studies had equal numbers of male and female participants; however, some studies such as McGill et al. [10] had more male participants than female. De Carvalho and Callaghan [4] found males to have significantly stiffer low stiffness zones (quartile one) compared to females. This gives rise to the possibility that the inclusion of McGill et al.’s [10] mildly male-dominated results may inflate the stiffness values for their first quartile. However, De Carvalho and Callaghan [4] only found this difference in stiffness due to sex for the first quartile, suggesting single-sex-dominated cohorts’ results should not significantly alter the combined results for the other three quartiles. This is somewhat challenged by Shojaei et al. [14], a study excluded from the current review due to lacking extractable data, who found a significant effect of sex on passive spinal stiffness at 70% of participants’ total range, suggesting the third quartile may similarly be impacted by single sex cohorts. Furthermore, Gruevski and Callaghan [7] found no significant effect due to sex at 40% flexion and Kosmopoulos et al. [8] found no difference either, whereas Tennant et al. [17] did. As some studies did find a significant difference, it is likely that sex may have an impact on passive stiffness; however, it is not possible from the current studies to clearly identify the magnitude of this impact nor whether this is the case for all planes of movement.

A further potential source of variability is the age of participants. Despite attempts to minimise the effect of older age by limiting participant cohorts to having an average age under 60 years, it is still possible that age may produce some variability in the results. This is somewhat limited in the results of the studies reviewed however as the average ages of participants ranged from 21.0 to 30.3 years. There are also other potentially confounding variables such as the height or weight of participants; however, without studies specifically investigating the effects of these characteristics on passive spinal stiffness, it is difficult to predict what, if any, effect they have on the results collated in this study.

### Validity and reliability

As discussed, there are a variety of potential causes for variability between studies. One way to mitigate against this variability is to ensure the methodologies used are both adequately valid and reliable; however, only one of the studies [6] reviewed has used a methodology meeting these criteria. In addition to this, Fewster et al. [5] investigated the reliability of their methodology but not the validity. This lack of consideration of validity and reliability among most of the included studies is potentially problematic as it calls into question the confidence that can be had in the methods they use. Development of valid and reliable techniques for measurements of this nature in flexion, extension and axial rotation is therefore an important area for future study. This is to ensure that future research seeking to investigate passive spinal stiffness in this way can have confidence in their results.

Despite this, the values presented by these studies still hold value in the current review. By comparing these studies, confidence intervals were able to be created within which the true mean stiffness values can be predicted to exist. The variation in methodology and results between these studies also provides insight into which methodological elements may be important to consider should someone be designing a similar study.

### Strengths and limitations

The present study provides synthesised reference values for passive in vivo spinal stiffness in all cardinal planes of movement. This is the first study to do so, offering a significant contribution to the literature. Moreover, through detailed discussion, this review highlights potentially confounding methodological differences between the studies included. From this, readers can gain valuable methodological insights for integration into future studies investigating the construct of in vivo passive stiffness.

As with many reviews of this kind, due to included studies being limited to those written in English, there is the potential for publication bias that may limit the generalizability of the results found. Several studies were excluded from this review due to a lack of extractable data. This suggests there is likely relevant data that exists on this topic, but it has not been possible to use this data in this study, limiting the depth of synthesis possible. This is further exacerbated by the different methods used by some included studies to report their findings. There are several articles that have reported their findings in non-comparable (non-convertible) units, meaning it was not possible to use their results in the synthesis, again limiting the depth to which this topic can be studied.

A further limitation to this study was the method used to establish confidence intervals for each plane of movement and quartile. The standard deviation and standard error of the mean were calculated for the compared studies based on the means of each study; however, this does not consider the variance contained within each study. Although this was recognised, the data did not exist to account for this across the studies; however, the standard deviations reported by the studies are shown in the data extraction table where possible.

A deliberate limitation of this study was the choice to exclude computation, cadaveric and animal studies from the synthesis. As the focus of this study was on the in vivo stiffness of humans, they were not appropriate for inclusion; however, it would be beneficial for future studies to synthesise reference stiffness values for these different study formats.

## Conclusion

This study has synthesised passive in vivo through-range lumbar spine stiffness values for all three cardinal planes of movements. As discussed however, there are a variety of confounding factors that should be considered when interpreting or utilising these values in further research. Axial rotation was found to be the least thoroughly investigated movement with confidence intervals not being producible for the third and fourth quartiles of range. This therefore suggests this may be an area that would benefit from further research. Both flexion and axial rotation would both benefit from the development of validated and reliable techniques for their stiffness measurements. This should be prioritised before further research is conducted into these areas. There is also now the scope to use these reference values to help establish the validity of computational models for predicting passive in vivo spinal stiffness. As relative stiffness between movements contradicted those observed in vitro, future studies could explore the cause of such discrepancies.
